# Tobacco Use and Treatment among Cancer Survivors

**DOI:** 10.3390/ijerph17239109

**Published:** 2020-12-06

**Authors:** Chineme Enyioha, Graham W. Warren, Glen D. Morgan, Adam O. Goldstein

**Affiliations:** 1 Department of Family Medicine, University of North Carolina at Chapel Hill, Chapel Hill, NC 27599, USA; chineme_enyioha@med.unc.edu (C.E.); glen.morgan.phd@gmail.com (G.D.M.); 2 Department of Radiation Oncology, Medical University of South Carolina, Charleston, SC 29425, USA; warrengw@musc.edu

Tobacco use is causally associated with the risk of developing multiple health conditions, including over a dozen types of cancer, and is responsible for 30% of cancer deaths in the U.S [1,2]. Smoking by cancer patients and survivors causes adverse health outcomes [3]. Smoking at the time of a cancer diagnosis increases the risk of overall mortality by approximately 50% and cancer-related mortality by approximately 60% across cancer disease sites and treatments [3]. Smoking further increases the risk of developing a second primary cancer and has strong associations with increased toxicity from cancer treatment [3]. Almost half of patients diagnosed with cancer who smoke may either continue to smoke while in treatment or relapse after successfully quitting, both of which complicate cancer care [4]. The annual costs of additional cancer treatment incurred by continued smoking are conservatively estimated at USD 3.4 billion in the United States [5]. However, quitting smoking after a cancer diagnosis is associated with improved overall survival [2] as well as improved cancer control and quality of life [6,7,8,9,10,11,12,13,14]. Despite evidence that demonstrates the harms of smoking, large surveys have shown that while approximately 90% of oncologists ask about tobacco use and over 80% advise patients to quit, few provide assistance with quitting [15,16]. A survey of 58 National Cancer Institute Designated Cancer Centers in 2009 demonstrated that most physicians did not provide structured approaches to addressing tobacco use [17]. Few Cooperative Group clinical trials have assessed tobacco use, even though smoking can affect overall and cancer-related survival, which serve as the primary or secondary outcomes of clinical trial designs [18]. Predictive barriers to providing cessation support include a lack of time, education and resources [19,20], but, until recently, there was little national coordination of efforts specifically designed to implement clinical cessation initiatives for cancer patients in the United States.

Supported by evidence from the 2014 Surgeon General’s Report on the clinical effects of smoking on cancer treatment outcomes [3], the National Comprehensive Cancer Network developed smoking cessation guidelines and advocated their use as a standard part of cancer care across all cancer disease sites [21]. Addressing tobacco use in oncology care and research has been advocated by several large cancer organizations [22,23,24,25,26]. To address the evidence base for the effects of smoking on cancer care and address the gap in the provision of smoking cessation support, the NCI introduced the Cancer Center Cessation Initiative (C3I) to provide financial and experiential support to enable cancer centers to implement evidence-based and sustainable tobacco use treatment programs [27]. From 2017 to 2019, 42 NCI Cancer Centers received funding and participated in structured network meetings that were coordinated through the C3I Coordinating Center at the University of Wisconsin [27]. In the C3I Initiative, centers were expected to develop individualized institutional programs that apply a population- and system-based approach to screen all cancer patients, offer evidence-based cessation support where appropriate, and report reach and effectiveness, with expected improvements in performance during the funding period.

It is in this context that the Special Issue on Tobacco Use and Treatment among Cancer Survivors of the *International Journal of Environmental Research and Public Health* makes a significant contribution to emerging research. This Special Issue focuses on identifying and understanding aspects of cancer care as it relates to tobacco use by highlighting the current research on tobacco use treatment and cessation for patients and survivors of cancer. The articles in this Special Issue address multiple aspects of tobacco use treatment for patients across the cancer spectrum, the development and integration of tobacco use treatment and services into cancer centers, the identification of barriers and challenges in existing tobacco use treatment programs, barriers to optimizing screening and referral systems, expansion of the availability of telemedicine, treatment for cancer patients in rural clinics, the integration of family members into tobacco use treatment to increase engagement, and the impact of the COVID-19 pandemic on tobacco use.

One major aspect of the screening and referral process that is addressed by multiple articles in this Special Issue involves strategies to improve referrals of cancer patients who smoke to tobacco use treatment by using an “opt-out” method, in which patients who screen positive for tobacco use are automatically referred for treatment [28]. Patients who are not interested can decline an appointment. This is opposite to the more common “opt-in” method, in which patients who screen positive are asked if they are open to a referral to a tobacco treatment program before a referral is made. Davis et al. [29] compared three methods for referring patients of a cancer center to a smoking cessation program in a retrospective observational study that compared traditional referral with a best practices alert (BPA) system and a pilot testing of direct outreach. Traditional referral was defined as unassisted referral by a provider who thought the patient would benefit from the services of a smoking cessation center. BPA involved an alert in the electronic medical record that reminded providers that a patient could benefit from a particular service or treatment. Direct outreach involved contacting eligible patients by phone to offer smoking cessation services. The findings from this study revealed that direct outreach had higher rates of referrals to and utilization of the cessation program compared with the other methods, and traditional referral had higher rates of program utilization compared with BPA. May and colleagues [30] evaluated the effectiveness of a patient-reported outcomes (PRO) tobacco use screener and automated referral system using a descriptive program evaluation design. The system was implemented through the patient portal of the electronic health record system of a cancer center and occurred concurrently with the launching of a provider-led referral system. Three times as many referrals were made through the PRO system compared with the provider-led referrals (164 vs. 59). Of the patients referred via the PRO system, 87% were contacted, and 43% of these engaged in treatment. Jose et al. [31] describe an EHR-based program to automatically refer cancer patients to tobacco use treatment using the “opt-out” system. This involved the design and delivery of a best practice advisory (BPA) system in the EHR that prompted the placement of a referral and scheduling of an appointment for tobacco treatment without the involvement of the provider and without information about the cessation plans or intentions of current smokers. After the implementation of the automatic referral system, a total of 210 referrals were placed by rooming staff, of which 71% had an appointment scheduled and 25% completed their appointment during the 6-week period of evaluation.

Despite improved referral services, in many cases, a minority of patients that use tobacco still enter treatment. Such findings were emphasized in the research by Sheffer and colleagues [32], who evaluated implementation and treatment outcomes in the first year of the development of a tobacco treatment service for cancer survivors. The authors describe the new screening and referral process and characterize the cancer survivors who agreed to treatment. Over the course of 15 months, while 5383 patients were referred for tobacco treatment, only 6.4% agreed to treatment and only 4% attended one treatment session or more. These studies highlight the fact that relying on traditional referral to cessation programs is insufficient and that direct outreach leads to an increase in referrals and enhances the utilization of smoking cessation centers’ services. Clearly, the feasibility of an EHR-based opt-out system of referring cancer patients to tobacco use treatment should become more prevalent across cancer centers. The inclusive “opt-out” method has been found to be beneficial in other patient populations in terms of increased referrals and treatment engagement [33,34]. Challenges clearly remain, however, in engaging increased numbers of referred patients with the completion of intensive treatment.

Several researchers conducted work to further examine this engagement issue. LeLaurin and colleagues [35] conducted a clinical trial to determine patient preferences, the acceptability of treatment, and the effectiveness of three tobacco treatment options among cancer patients. The treatment options included a 6-week cognitive behavioral therapy (CBT) program that was delivered through a smartphone teleconference (SmartQuit), referral to external services such as the quitline (PhoneQuit), and in-person group counseling (GroupQuit). A total of 90 patients were enrolled in the study: 16% in GroupQuit, 43% in PhoneQuit, and 41% in SmartQuit. Of the 35 patients who were reached at the 12-week follow-up, 54% noted the receipt of tobacco treatment. At the 12-week follow-up, the rate of abstinence was higher among those who used GroupQuit, and the SmartQuit intervention had higher acceptability compared with the other two groups. This study raises the question of whether tailored implementation strategies may improve engagement.

Similarly, Gali and colleagues [36] attempted to improve treatment referrals and engagement by addressing common barriers to treatment. Their process involved an opt-out system to maximize screening and referrals, the delivery of treatment via telemedicine, and the same-day delivery of medication, in addition to individual counseling to maximize engagement. Treatment was free of charge for patients who received care. Two hundred and seventy-three patients were reached by phone, and 33% engaged in treatment. The program was expanded to other clinics, and after one year, a total of 14 clinics were providing free tobacco treatment.

Ramsey and colleagues [37] examined the treatment engagement of cancer patients in urban and rural settings with an EHR-based, low-burden tobacco cessation module by measuring patients’ acceptance of treatment. The prevalence of smoking was higher and treatment engagement was lower in patients who received care in rural clinics. Across sites, patients in clinics that implemented the cessation module were more likely to receive treatment compared with patients in clinics that did not implement the module. This study is relevant to efforts that might increase engagement, particularly in settings with fewer resources.

Another major advance in quality improvement that is discussed in this Special Issue is the application of Lean Six Sigma principles to tobacco use treatment. Wiseman and colleagues [38] conducted a study to evaluate the processes of assessing and documenting tobacco use among cancer survivors and the referral to resources for tobacco use treatment. Using the five principles of Lean methodology, 11 personnel and nine EHR gaps were identified. Addressing inconsistency in the documentation of tobacco use in the EHR was identified as the top priority. Prioritized gaps were addressed with modifications to the EHR, changes to the workflow, and training of providers. A new tobacco treatment program was implemented based on the changes made, and the results showed that the number of patients who received cessation support increased from 284 in the first year to 487 in the third year of the new program. This study demonstrates the interdependence of various elements involved in patient care and how to address gaps that can improve cessation outcomes. Meyer et al. [39] applied Lean Six Sigma principles to expose and improve inefficiencies in the workflow of a tobacco treatment program. The team used a step-by-step approach related to the workflow and patient care that began with creating a “reason for action”, followed by mapping the current and target states. New patient referrals and counseling sessions were the two metrics of focus. The team focused on four gaps and targeted their experiments to address those gaps. The gaps were low program awareness, inconsistency in the referral process, low rate of referral from an affiliated department, and the burden associated with data collection. After a 12-month period, the mean numbers of new patient referrals and counseling sessions increased by 140% and 13%, respectively. The findings from this study show that a targeted approach to addressing gaps in tobacco treatment programs can lead to positive outcomes.

Establishing and implementing sustainable tobacco use treatment programs in cancer centers has been a major goal of the NCI, and to do so in comprehensive organizations such as cancer centers may require systems changes within academic medical organizations. Tong et al. [40] described a new tobacco treatment program that was developed using a systems approach. The group adapted domains of the Cancer Care Continuum as a systems framework to identify areas of need and applied constructs from the Consolidated Framework of Implementation Research to describe the implementation process. The processes resulted not only in referral to a quitline or group classes but also in motivational quality improvement drivers at the health system level and mandated accreditation programs such as the American College of Surgeon’s Commission on Cancer. They also assessed the implementation readiness of the cancer center based on different domains of the Cancer Care Continuum; implementation readiness was lacking in domains of screening, diagnosis, treatment, and survivorship. The findings from this study highlight the importance of systematic thinking to identify and address challenges associated with integrating a tobacco treatment program into a cancer center.

Several researchers have published reports on unique issues in tobacco use and cancer care. Ruebush et al. [41] examined the feasibility of the systematic integration of family members into the patient’s tobacco treatment program in a cancer hospital and the impact of such integration on quit rates. The 18-month pilot study entailed modification of the EHR, training of tobacco treatment specialists on family counseling, integration of family members into patient treatment, and follow-up with patients at 6 months to analyze quit rates. The authors found that of 532 enrolled patients who received tobacco treatment, 42% had family members integrated into their treatment, and almost all others did not have family members around during treatment. The quit rate at 6 months was 28% for patients who had family members as part of their treatment and 23% for those without family members involved, but the difference was not statistically significant. The findings from this study suggest that integrating family members into tobacco treatment for patients is a feasible and promising intervention for further study.

The overwhelming majority of evidence related to smoking in cancer involves combustible cigarettes, with far less information on other forms of tobacco consumption. The study by Kowitt et al. is unique, as it examined cigar smokers and their perception of risks from COVID-19, their quit intentions, and their behaviors during the COVID-19 pandemic. Among a survey of 777 cigar smokers, 76% had a greater perception of risk of suffering complications from COVID-19 compared with non-smokers. While the majority of participants noted an intention to quit as a result of COVID-19, more participants reported increases in smoking since the onset of the pandemic. Given the markedly increased risks that cigar users have of cancers of the larynx and oral cavity, this research is timely and can help inform more effective interventions for at-risk cigar users.

This Special Issue reflects the diverse aspects of addressing tobacco use treatment as an essential part of cancer care. Importantly, each manuscript highlights the diversity of approaches that can be used and emphasizes the need to consider evidence-based care in a manner that can be implemented and sustained based upon institutional needs and resources. Although many barriers still exist at various levels, from the patient level to clinic or health system levels, this Special Issue describes ways in which some of these barriers can be mitigated to improve care. Overcoming some of the identified barriers will involve fairly simple modifications to existing processes or the introduction of a novel step. By expanding reach and access to care, the integration of effective tobacco use treatment programs into cancer centers or clinics will lead to improved outcomes and a reduction in smoking-related health consequences among cancer patients.

## References

[1] Jacobs E.J., Newton C.C., Carter B.D., Feskanich D., Freedman N.D., Prentice R.L., Flanders W.D. (2015). What proportion of cancer deaths in the contemporary United States is attributable to cigarette smoking?. Ann. Epidemiol..

[2] U.S. Department of Health and Human Services (2020). Smoking Cessation: A Report of the Surgeon General.

[3] U.S. Department of Health and Human Services (2014). The Health Consequences of Smoking–50 Years of Progress: A Report of the Surgeon General.

[4] Lucchiari C., Masiero M., Botturi A., Pravettoni G. (2016). Helping patients to reduce tobacco consumption in oncology: A narrative review. Springerplus.

[5] Warren G.W., Cartmell K.B., Garrett-Mayer E., Salloum R.G., Cummings K.M. (2019). Attributable Failure of First-line Cancer Treatment and Incremental Costs Associated With Smoking by Patients With Cancer. JAMA Netw. Open.

[6] Schild S.E., Tan A.D., Wampfler J.A., Ross H.J., Yang P., Sloan J.A. (2015). A new scoring system for predicting survival in patients with non-small cell lung cancer. Cancer Med..

[7] Dobson Amato K.A., Hyland A., Reed R., Mahoney M.C., Marshall J., Giovino G., Bansal-Travers M., Ochs-Balcom H.M., Zevon M.A., Cummings K.M. (2015). Tobacco Cessation May Improve Lung Cancer Patient Survival. J. Thorac. Oncol. Off. Publ. Int. Assoc. Study Lung Cancer.

[8] Al-Mamgani A., van Rooij P.H., Mehilal R., Verduijn G.M., Tans L., Kwa S.L.S. (2014). Radiotherapy for T1a glottic cancer: The influence of smoking cessation and fractionation schedule of radiotherapy. Eur. Arch. Oto-Rhino-Laryngol..

[9] Passarelli M.N., Newcomb P.A., Hampton J.M., Trentham-Dietz A., Titus L.J., Egan K.M., Baron J.A., Willett W.C. (2016). Cigarette Smoking Before and After Breast Cancer Diagnosis: Mortality From Breast Cancer and Smoking-Related Diseases. J. Clin. Oncol..

[10] Parsons A., Daley A., Begh R., Aveyard P. (2010). Influence of smoking cessation after diagnosis of early stage lung cancer on prognosis: Systematic review of observational studies with meta-analysis. BMJ (Clin. Res. Ed.).

[11] Garces Y.I., Yang P., Parkinson J., Zhao X., Wampfler J.A., Ebbert J.O., Sloan J.A. (2004). The relationship between cigarette smoking and quality of life after lung cancer diagnosis. Chest.

[12] Zhao S., Chen F., Wang D., Wang H., Han W., Zhang Y. (2019). Effect of preoperative smoking cessation on postoperative pain outcomes in elderly patients with high nicotine dependence. Medicine.

[13] Lugg S.T., Tikka T., Agostini P.J., Kerr A., Adams K., Kalkat M.S., Steyn R.S., Rajesh P.B., Bishay E., Thickett D.R. (2017). Smoking and timing of cessation on postoperative pulmonary complications after curative-intent lung cancer surgery. J. Cardiothorac. Surg..

[14] Quan H., Ouyang L., Zhou H., Ouyang Y., Xiao H. (2019). The effect of preoperative smoking cessation and smoking dose on postoperative complications following radical gastrectomy for gastric cancer: A retrospective study of 2469 patients. World J. Surg. Oncol..

[15] Warren G., Marshall J., Cummings K., Toll B., Gritz E., Hutson A., Dibaj S., Herbst R., Dresler C. (2013). IASLC Tobacco Control and Smoking Cessation Committee. Practice patterns and perceptions of thoracic oncology providers on tobacco use and cessation in cancer patients. J. Thorac. Oncol..

[16] Warren G.W., Marshall J.R., Cummings K.M., Toll B.A., Gritz E.R., Hutson A., Dibaj S., Herbst R., Mulshine J.L., Hanna N. (2013). Addressing tobacco use in patients with cancer: A survey of American Society of Clinical Oncology members. J. Oncol. Pract..

[17] Goldstein A.O., Ripley-Moffitt C.E., Pathman D.E., Patsakham K.M. (2013). Tobacco use treatment at the U.S. National Cancer Institute’s designated Cancer Centers. Nicotine Tob. Res..

[18] Peters E.N., Torres E., Toll B.A., Cummings K.M., Gritz E.R., Hyland A., Herbst R.S., Marshall J.R., Warren G.W. (2012). Tobacco assessment in actively accruing national cancer institute cooperative group program clinical trials. J. Clin. Oncol..

[19] Wells M., Aitchison P., Harris F., Ozakinci G., Radley A., Bauld L., Entwistle V., Munro A., Haw S., Culbard B. (2017). Barriers and facilitators to smoking cessation in a cancer context: A qualitative study of patient, family and professional views. BMC Cancer.

[20] Warren G.W., Dibaj S., Hutson A., Cummings K.M., Dresler C., Marshall J.R. (2015). Identifying Targeted Strategies to Improve Smoking Cessation Support for Cancer Patients. J. Thorac. Oncol..

[21] Price S.N., Studts J.L., Hamann H.A. (2019). Tobacco Use Assessment and Treatment in Cancer Patients: A Scoping Review of Oncology Care Clinician Adherence to Clinical Practice Guidelines in the U.S. Oncologist.

[22] Hanna N., Mulshine J., Wollins D.S., Tyne C., Dresler C. (2013). Tobacco cessation and control a decade later: American Society of Clinical Oncology policy statement update. J. Clin. Oncol..

[23] Land S.R., Toll B.A., Moinpour C.M., Mitchell S.A., Ostroff J.S., Hatsukami D.K., Duffy S.A., Gritz E.R., Rigotti N.A., Brandon T.H. (2016). Research priorities, measures, and recommendations for assessment of tobacco use in clinical cancer research. Clin. Cancer Res..

[24] Toll B.A., Brandon T.H., Gritz E.R., Warren G.W., Herbst R.S. (2013). Assessing tobacco use by cancer patients and facilitating cessation: An American Association for Cancer Research policy statement. Clin. Cancer Res..

[25] Cummings K.M., Dresler C.M., Field J.K., Fox J., Gritz E.R., Hanna N.H., Ikeda N., Jassem J., Mulshine J.L., Peters M.J. (2014). E-cigarettes and cancer patients. J. Thorac. Oncol..

[26] Sarna L., Bialous S.A. (2004). Tobacco control policies of oncology nursing organizations. Semin. Oncol. Nurs..

[27] Croyle R.T., Morgan G.D., Fiore M.C. (2019). Addressing a Core Gap in Cancer Care—The NCI Moonshot Program to Help Oncology Patients Stop Smoking. N. Engl. J. Med..

[28] Richter K.P., Ellerbeck E.F. (2015). It’s time to change the default for tobacco treatment. Addiction.

[29] Davis J.M., Thomas L.C., Dirkes J.E.H., Swartzwelder H.S. (2020). Strategies for Referring Cancer Patients in a Smoking Cessation Program. Int. J. Environ. Res. Public Health.

[30] May J.R., Klass E., Davis K., Pearman T., Rittmeyer S., Kircher S., Hitsman B. (2020). Leveraging Patient Reported Outcomes Measurement via the Electronic Health Record to Connect Patients with Cancer to Smoking Cessation Treatment. Int. J. Environ. Res. Public Health.

[31] Jose T., Ohde J.W., Hays J.T., Burke M.V., Warner D.O. (2020). Design and Pilot Implementation of an Electronic Health Record-Based System to Automatically Refer Cancer Patients to Tobacco Use Treatment. Int. J. Environ. Res. Public Health.

[32] Sheffer C.E., Stein J.S., Petrucci C., Mahoney M.C., Johnson S., Giesie P., Carl E., Krupski L., Tegge A.N., Reid M.E. (2020). Tobacco Dependence Treatment in Oncology: Initial Patient Clinical Characteristics and Outcomes from Roswell Park Comprehensive Cancer Center. Int. J. Environ. Res. Public Health.

[33] Campbell K.A., Bowker K.A., Naughton F., Sloan M., Cooper S., Coleman T. (2016). Antenatal Clinic and Stop Smoking Services Staff Views on “Opt-Out” Referrals for Smoking Cessation in Pregnancy: A Framework Analysis. Int. J. Environ. Res. Public Health.

[34] Buchanan C., Nahhas G.J., Guille C., Cummings K.M., Wheeler C., McClure E.A. (2017). Tobacco Use Prevalence and Outcomes Among Perinatal Patients Assessed Through an “Opt-out” Cessation and Follow-Up Clinical Program. Matern. Child Health J..

[35] LeLaurin J.H., Dallery J., Silver N.L., Markham M.-J., Theis R.P., Chetram D.K., Staras S.A., Gurka M.J., Warren G.W., Salloum R.G. (2020). An Implementation Trial to Improve Tobacco Treatment for Cancer Patients: Patient Preferences, Treatment Acceptability and Effectiveness. Int. J. Environ. Res. Public Health.

[36] Gali K., Pike B., Kendra M.S., Tran C., Fielding-Singh P., Jimenez K., Mirkin R., Prochaska J.J. (2020). Integration of Tobacco Treatment Services into Cancer Care at Stanford. Int. J. Environ. Res. Public Health.

[37] Ramsey A.T., Baker T.B., Pham G., Stoneking F., Smock N., Colditz G.A., James A.S., Liu J., Bierut L.J., Chen L.-S. (2020). Low Burden Strategies Are Needed to Reduce Smoking in Rural Healthcare Settings: A Lesson from Cancer Clinics. Int J. Env. Res. Public Health.

[38] Wiseman K.P., Hauser L., Clark C., Odumosu O., Dahl N., Peregoy J., Sheffield C.W., Klesges R.C., Anderson R.T. (2020). An Evaluation of the Process and Quality Improvement Measures of the University of Virginia Cancer Center Tobacco Treatment Program. Int. J. Environ. Res. Public Health.

[39] Meyer C., Mitra S., Ruebush E., Sisler L., Wang K., Goldstein A.O. (2020). A Lean Quality Improvement Initiative to Enhance Tobacco Use Treatment in a Cancer Hospital. Int. J. Environ. Res. Public Health.

[40] Tong E.K., Wolf T., Cooke D.T., Fairman N., Chen M.S. (2020). The Emergence of a Sustainable Tobacco Treatment Program across the Cancer Care Continuum: A Systems Approach for Implementation at the University of California Davis Comprehensive Cancer Center. Int. J. Environ. Res. Public Health.

[41] Ruebush E., Mitra S., Meyer C., Sisler L., Goldstein A.O. (2020). Using a Family Systems Approach to Treat Tobacco Use among Cancer Patients. Int. J. Environ. Res. Public Health.

